# Peace and Goodwill

**Published:** 1887-12-24

**Authors:** 


### WORDS OF CONSOLATION.

[Communications for this column should be addressed "Chaplain," care of the Editor of The Hospital, who will gratefully welcome any suggestions or inquiries from matrons, nurses, and the staff generally.]

### "Peace and Goodwill."

It was a striking announcement made by beings belonging to other worlds as well as to this that a child about to be born in a little Judæan village should be the harbinger of "peace and goodwill toward men." All of us who have had much experience of life know what sweetness there is even in the words; and how much more restful still the actual presence of the "peace and goodwill" are. There are those who point scoffingly to the wars which still rend the world in pieces, and ask: "Where is your peace and goodwill?" We may leave the larger world to its own concerns for a brief while and find an evidence of the reality of both a little nearer home. Some who read this are lying on beds of suffering in hospital wards. Is not the ward in which you are lying a place of ordered and sure peace? If you have been in your present place for two or three weeks you have begun to feel quite satisfied that so long as you remain where you are you are assured that every day will be a day of quietness, of orderly behaviour, of restful peace. And do you not feel that you are living in an atmosphere of kindliness? And what is that but the fruit of "goodwill"? It is "goodwill" first on the part of those who had it in their hearts to build hospitals; then on the part of those who give the money for their building and maintenance; then on the part of the hard-working physicians and surgeons, who give their learning and their skill; then on the part of matrons, nurses, and all the other hospital officials. If these be not practical proofs of "goodwill" on the part of all sorts and conditions of men, we know not where to look for them. When, therefore, you read again in your New Testament the beautiful story of the birth of Jesus Christ, you need have no doubt that the "peace upon earth and goodwill among men," which were then announced, have been largely realised, and that as men grow wiser and more instructed, they will be much more universally realised in the days yet to come.

